# Optimization of resting tension for wire myography in male rat pulmonary arteries

**DOI:** 10.14814/phy2.15911

**Published:** 2024-01-11

**Authors:** Rira Choi, Roshini Narayanan, Sandeep Jandu, William Savage, Sara Kang, Bulouere Wodu, Kavitha Nandakumar, Lakshmi Santhanam, Jochen Steppan

**Affiliations:** ^1^ Department of Chemical and Biomolecular Engineering Johns Hopkins University Baltimore Maryland USA; ^2^ Department of Anesthesiology and Critical Care Medicine Johns Hopkins School of Medicine Baltimore Maryland USA; ^3^ Department of Biomedical Engineering Johns Hopkins University Baltimore Maryland USA; ^4^ Present address: Department of Biomedical Engineering Yale University New Haven Connecticut USA

**Keywords:** protocol, pulmonary arterial hypertension, pulmonary hypertension, remodeling, vascular stiffness, wire myography

## Abstract

Wire myography to test vasomotor functions of blood vessels ex‐vivo are well‐established for the systemic circulation, however, there is no consensus on protocols for pulmonary arteries. We created a standardized wire myography protocol for healthy rat PAs and validated this in a pulmonary hypertension (PH) model. Vessels stretched to higher initial tensions (5.0, 7.5 and 10.0 mN) exhibited a uniform response to phenylephrine, a larger dynamic range, and lower EC50 values. The endothelium‐mediated relaxation showed that moderate tensions (7.5 and 10.0 mN) produced robust responses with higher maximum relaxation and lower EC50 values. For endothelium independent responses, the higher initial tension groups had lower and more consistent EC50 values than the lower initial tension groups. Pulmonary arteries from rats with PH were more responsive to vasoactive drugs when subjected to a higher initial tension. Notably, vessels in the PH group subjected to 15.0 mN exhibited high dynamic ranges in contractile and relaxation responses without tearing. Lastly, we observed attenuated cholinergic responses in these vessels—consistent with endothelial dysfunction in PH. Therefore, a moderate initial tension of 7.5–10.0 mN is optimal for healthy rat pulmonary arteries and a higher initial tension of 15.0 mN is optimal for pulmonary arteries from animals with PH.

## 1 INTRODUCTION

Pulmonary diseases are among the most prevalent diseases worldwide, costing the lives of millions of people annually (Berman, 1991; Wang et al., 2016). In addition to the COVID‐19 pandemic, acute respiratory distress syndrome (ARDS), chronic obstructive pulmonary disease (COPD), and pulmonary arterial hypertension (PAH) cause significant pulmonary inflammation, inflicting irreversible damages and structural remodeling of the lung vasculature (Revercomb et al., 2020; Shah et al., 2021; Wang et al., 2021). PAH, for example, progressively and irreversibly alters the function of the pulmonary arteries, leading to right ventricular failure (Thompson & Lawrie, 2017). While the pathological pathways that drive the development of PAH are not completely understood, increased pulmonary vascular resistance in PAH is generally associated with functional and structural changes in the pulmonary vasculature (Steppan et al., 2016). Aberrant changes in the vasculature of patients with PAH are caused by a myriad of cellular and molecular pathways. Importantly, vasoconstriction does not necessarily serve as the sole cause of PAH, but can actually be its consequence (Martin et al., 2006). In this context it is interesting to note that it is increasingly recognized that the proximal PAs, which are the major determinant PA stiffness are indeed the main determinant of right ventricular impedance and cushion pulsatile flow, directly influencing right ventricular afterload via ventricular to arterial coupling, while the distal vasculature is more representative of pulmonary vascular resistance (Sun & Chan, 2018). Thus, the structural and functional changes noted in PAH are caught in a vicious positive feedback loop with vasoconstriction causing structural changes, and the structural changes in turn, leading to vasoconstriction. Similarly, the pulmonary arteries in patients with COPD, ARDS, and pulmonary fibrosis demonstrate significant structural changes (Chaouat et al., 2008; Eskandari et al., 2013; Ryan et al., 2014). It is, therefore, of critical importance to characterize pulmonary vascular function in multiple different disease models to develop targeted therapies.

In this context, in vitro wire myography is of particular interest as it precisely measures the vasoactive responses of isolated vessels to targeted pharmacological interventions in real‐time, and can be applied to a variety of animal species and in a multitude of disease models (Mulvany & Aalkjaer, 1990). Wire myography experiment protocols have been well‐established and readily applied in studies involving different vessel types of the systemic circulation from large compliance vessels such as the aorta to small resistance vessels such as the coronary artery (Hori et al., 2017; Kristiansen et al., 2017; Sikka et al., 2014; Steppan et al., 2017, 2019; Steppan, Jandu, Savage, et al., 2020; Steppan, Jandu, Wang, et al., 2020). When using rat aorta, it is common practice to stretch the vessel to 15.0 mN (Soucy et al., 2010). However, different vascular beds necessitate different levels of preconstriction due to the baseline in‐vivo pressure differences for each system. For rat basilar arteries and rat coronary arteries, the optimal initial tensions of the vessel segments for a wire myography experiment were empirically determined to be 1.63 ± 0.01 and 1.16–1.52 mN/mm respectively (Ping et al., 2014; Xiao et al., 2015). Overall, wire myography involving systemic vessels have been optimized and reliably established through empirical and literary conventions (Bridges et al., 2011; Ishida et al., 2019; Mulvany & Aalkjaer, 1990). On the contrary, there is a distinct lack of consensus on the optimal stretch conditions for pulmonary arteries, which are exposed to significantly lower pressures in situ (Alvarez et al., 2017; Chan et al., 2005; Ko et al., 2010; Nyhan et al., 2002).

The maximum active tension development is achieved when the vessel segments are stretched to a normalized internal circumference (ICn) that mimics physiological condition with the transmural pressure specified at 100 mmHg (IC100) by a factor of 0.9: (Konja et al., 2019; Ping et al., 2014; Xiao et al., 2015).

ICn=0.9·IC100

This approach, to normalize against the transmural pressure specified at 100 mmHg, is reasonable for vessels of the systemic circulation that have high pressure gradients in vivo. However, the pressure gradient from the pulmonary arteries to the left atrium is only approximately 10 mmHg (Widrich & Shetty, 2023). Therefore, the pre‐stretch values obtained using the aforementioned standard are questionable for low resistance vessels such as the pulmonary arteries. Furthermore, this normalization protocol is designed specifically for small arterial resistance vessels.

An alternative approach to determine optimal prestretch for wire myography is to use the minimum level of stretch value that allowed for the largest contractile response to KCl as the optimal resting tension. While this method has been adopted and cited, different groups use different tension values ranging from 0.98–5.88 mN (Alvarez et al., 2017; Chan et al., 2005; Ko et al., 2010; Nyhan et al., 2002). Such discrepancies of optimal resting tension for pulmonary arteries are concerning, as inconsistencies in standardization prevent not only the acquisition of reliable data but also the comparison between datasets.

Therefore, the goal of this study was to develop a standardized protocol for pulmonary arteries and to test its application in a disease model with significant pulmonary vascular disease. We used the monocrotaline (MCT) rat model of pulmonary hypertension given its wide use and its reliability in accurately mimicking human pulmonary vascular disease. Animals exposed to monocrotaline develop severe pulmonary hypertension and high pulmonary artery pressures (Bueno‐Beti et al., 2018; Sun & Chan, 2018).

## 2 MATERIALS AND METHODS

### 2.1 Animals used

A total of 40 male Wistar rats (weight 150–175 g) were purchased from Charles River (Wilmington, MA). The number of animals used for each group was *n* = 22 for the healthy group, and *n* = 18 for the PAH group. All animals were maintained in the Johns Hopkins University School of Medicine animal care facility. All protocols were approved by Institutional Animal Care and Use Committee. The animals were fed and watered ad libitum and maintained on a 12‐hour light/dark cycle.

### 2.2 Monocrotaline induced pulmonary arterial hypertension (PAH)

PAH was induced by a single subcutaneous injection of 60 mg/kg of monocrotaline (MCT; Cayman Chemical, Ann Arbor, Michigan, catalogue number: 16666) in the ventral abdomen. Right ventricular systolic pressures as a surrogate of pulmonary arterial pressures were determined using pressure volume loops a 1.4‐F SPR‐839 Millar conductance catheter (Millar, Inc, Houston, TX) that was introduced into the right ventricle via the apex of the heart. Data was recorded with a Millar Aria 1 PV Conductance System and Chart 5 from AD Instrument PowerLab (AD instruments, Colorado Springs, CO). Occlusion of the inferior vena cava resulted in a transient reduction in preload that was used to obtain pressure volume loops and chamber‐specific hemodynamic variables.

### 2.3 Calibration of the force transducer unit

The force applied by the vessel walls on the pin is measured in units of milligram‐Force (mg‐Force). The mg‐Force measurement is then converted to mN by the myo‐interface (Danish Myo Technology, MI; Multi Myograph System 620 M). The system is calibrated using a 2‐point calibration (0 and 1000 mg‐Force; 0 and 9.81 mN) following the manufacturer's protocol.

### 2.4 Isolation of pulmonary arteries

The heart‐lung block is isolated under a microscope by removing the connective tissues, excising the aorta, pulmonary veins, and trachea. The first left and right branch of the pulmonary artery are isolated, cut into 2 mm rings, and mounted onto the pins located in the chamber of the wire myography unit (Danish Myo Technology, MI). Each chamber is filled with 6 mL of Krebs–Henseleit buffer solution (containing 118 mM NaCl, 4.7 mM KCl, 2.5 mM CaCl_2_∙H_2_O, 1.1 mM KH_2_PO_4_, 25 mM NaHCO_3_, 1.2 mM MgSO_4_, and 11 mM glucose at a pH of 7.4), and continuously bubbled with 95% O_2_ and 5% CO_2_ at 37°C. The dimensions of the arteries are measured by taking digital photographs of the cross‐sections of each vessel segment using a light microscope.

### 2.5 Vessel pre‐stretch

The vessels are not exposed to extreme mechanical stress which may lead to compromised structural integrity and loss of physiological function (Table 1). The incremental values are determined in such a manner as to minimize the number of pre‐stretch steps required to achieve the intended final tension. Between each stretch, the vessels equilibrate for 5‐minutes. After the final pre‐stretch, the vessels are equilibrated for 20 min.

**TABLE 1:** Pre‐stretch groups.

	Pre‐stretch #1	Pre‐stretch #2	Pre‐stretch #3	Pre‐stretch #4	Pre‐stretch #5	Pre‐stretch #6	Pre‐stretch #7	Pre‐stretch #8	Pre‐stretch #9
	(mN)	(mN)	(mN)	(mN)	(mN)	(mN)	(mN)	(mN)	(mN)
Condition 1 (Tension 15 mN)	0.5	1.0	2.25	3.5	5.0	7.5	10.0	12.5	**15.0**
Condition 2 (Tension 10.0 mN)	**–**	**–**	0.5	1.0	2.25	3.5	5.0	7.5	**10.0**
Condition 3 (Tension 7.5 mN)	**–**	**–**	**–**	0.5	1.0	2.25	3.5	5.0	**7.5**
Condition 4 (Tension 5.0 mN)	**–**	**–**	**–**	**–**	0.5	1.0	2.25	3.5	**5.0**
Condition 5 (Tension 2.5 mN)	**–**	**–**	**–**	**–**	0.25	0.5	1.0	1.5	**2.5**
Condition 6 (Tension 1.0 mN)	**–**	**–**	**–**	**–**	**–**	0.25	0.5	0.75	**1.0**
Condition 7 (Tension 0.5 mN)	**–**	**–**	**–**	**–**	**–**	**–**	**–**	0.25	**0.5**

*Note*: Accumulated value of force applied to the vessel segments at each pre‐stretch step.

Table 1 depicts the experimental groups, demonstrating the number and amount of stretch applied until final tension (bold) was reached.

### 2.6 Vessel viability and pharmacological dose responses

Each isolated vessel ring is subjected to only a single pre‐stretch tension per experiment. The viability of each vessel segment is tested using potassium chloride (KCl; 60 mM, Sigma‐Aldrich, catalogue number: P3911) (Wenceslau et al., 2021), followed by a 45 min stabilization period. After washing, vasocontractile response is determined with increasing concentrations of PE (10^−9^ to 10^−5^ mol/L; Sigma‐Aldrich, catalogue number: P1240000). Data are normalized to the prior KCl response:

$$\begin{array}{c} \text{Response Normalized to}\;\mathrm{KCl}\;\left(\%\right)\\ \;=\frac{\mathrm{PE}\;\text{Response}-\text{Baseline}}{\text{Maximum}\;\mathrm{KCl}\;\text{response}}\times 100. \end{array}$$

After washing, the pulmonary arteries are pre‐constricted with phenylephrine (10^−6^ mol/L; Sigma Aldrich, catalogue number: P1240000) and stabilize for 30 minutes. Following this PE pre‐constriction, either endothelium‐dependent or ‐independent vasorelaxation responses are determined with acetylcholine (10^−9^ to 10^−5^ mol/L; Sigma‐Aldrich, catalogue number: A6625) or sodium nitroprusside (10^−9^ to 10^−5^ mol/L; Sigma‐Aldrich, catalogue number: 71778) respectively. The relaxation data are normalized to the PE pre‐constriction value as follows:

$$\begin{array}{c} \text{Percent relaxation}\;\left(\%\right)=\left(\frac{\text{Maximum}\;\mathrm{PE}\;\text{Constriction}-\mathrm{Ach}\;\text{or}\;\mathrm{SNP}\;\text{Remaining Constriction}}{\text{Maximum}\;\mathrm{PE}\;\text{Constriction}-\text{Baseline}}\right)\times 100. \end{array}$$

Additionally, the dynamic range of the vascular response to PE, Ach, and SNP are calculated by subtracting the minimum horizontal asymptote from the maximum horizontal asymptote from the dose response curves.

### 2.7 Vessel dimensions

After dissection, vessels were placed on a light microscopy stage (Laxco LMC4‐BF155, BF Binoc Microscope, Laxco, Mill Creek, WA) and we obtained transverse images of each sample at 4× magnification to calculate vessel dimensions [internal lumen diameter, external lumen diameter, and wall thickness], using SeBaview Software (Laxco, Mill Creek, WA).

### 2.8 Data collection and analysis

Initial tension levels are randomly assigned to different wire myograph chambers each time to eliminate sources of bias originating from acquisition of tension data in the same myograph chamber. Replicate data were tested for normality using GraphPad Prism (GraphPad Software, San Diego, CA), and an unpaired Student's *t*‐test was used to compare two means. Analysis of variance (ANOVA) tests were performed to determine statistical significance when comparing multiple data sets. Bonferroni post hoc analysis was used for multiple comparisons, data are represented as mean ± standard deviation, and statistical significance was set at *p* < 0.05.

## 3 RESULTS

### 3.1 Induction of pulmonary hypertension

Rats in the pulmonary hypertension group had significantly higher pulmonary artery pressures compared to control animals (60.0 vs. 26.8 mmHg, *n* = 8, *p* = 0.005).

### 3.2 Vessel dimensions

**FIGURE 1:**
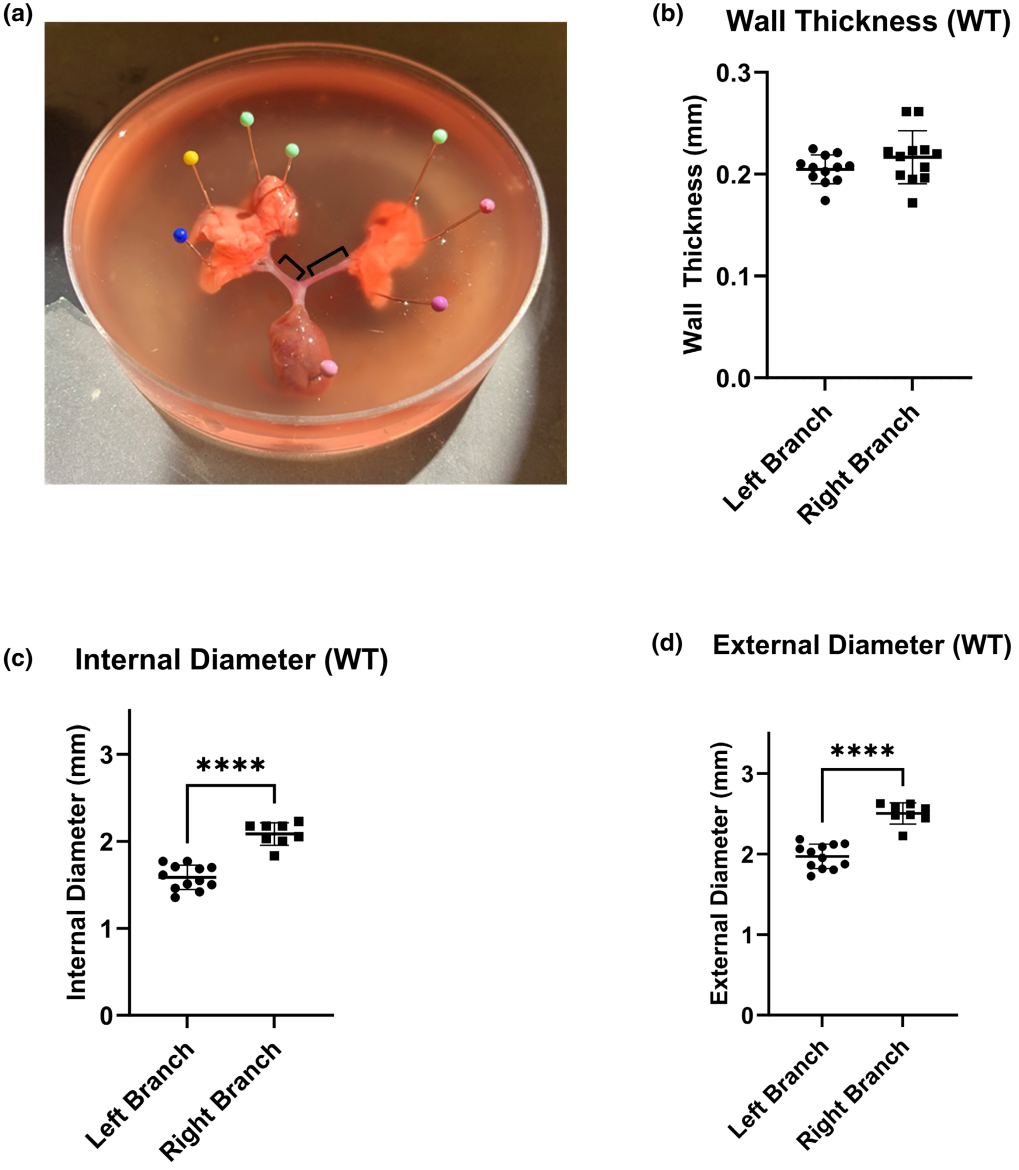
Vessel diameters and thickness. (a) Rat heart lung block from which the primary left and right PA branches (indicated by black lines) were removed for the experiments. (b) There was no difference between the wall thicknesses of the vessel segments (*n* = 8). External (c) and internal (d) diameters of the vessel segments from the right branch were larger than the left. (****p < 0.0001 by unpaired Student's *t*‐test).

A representative dissection of the isolated PAs is depicted in Figure 1a. Average vessel wall thickness of the pulmonary artery was determined to be 0.21 ± 0.03 mm for the left branch and 0.22 ± 0.06 mm for the right branch (Figure 1b; *p* = 0.18; *n* = 8 each). Internal lumen diameters (Figure 1c) were 1.59 ± 0.12 mm for the left branch and 2.08 ± 0.09 mm for the right branch (*p* < 0.0001). The average external diameters were 1.97 ± 0.11 mm for the left branch and 2.50 ± 0.08 mm for right branch (*p* < 0.0001, Figure 1d).

### 3.3 Pre‐stretch and vessel viability

**FIGURE 2:**
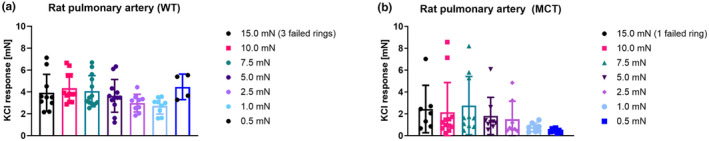
Maximum contraction response of pulmonary arteries. (a) This figure demonstrates the maximum contractile response of each healthy vessel stretched to a different initial tension. Higher levels of preconstruction lead to an increased number of mechanical vessel failures, most prominent at 15.0 mN. (b) This figure depicts the maximum contractile response observed in rat pulmonary arteries with PAH.

Healthy vessels stretched to 15.0 mN exhibited lower maximum KCl constriction values than vessels pre‐stretched to 10.0 or 7.5 mN (Figure 2a), with a significant number of vessels tearing apart under 15.0 mN (these vessels were removed from the KCl analysis). Given the high rate of vessel failure and the poor and variable KCl response, the 15.0 mN initial tension group was excluded from further studies for healthy PAs. All remaining groups showed a comparable maximum contractile response with no statistically significant difference between the groups (*p* = 0.580). In PAs from animals with PAH, 15.0 mN pre‐stretch produced a robust and reliable response. Thus, 15.0 mN was included for further analysis in the MCT group. While healthy PAs on average exhibited higher maximum contractile responses to KCl across all tension groups, MCT PAs had a blunted response to KCl with the exception of the 15.0 mN group being most responsive to KCl (1.67 mN; Figure 2b).

### 3.4 Initial tension effects on vasoconstriction response

**FIGURE 3:**
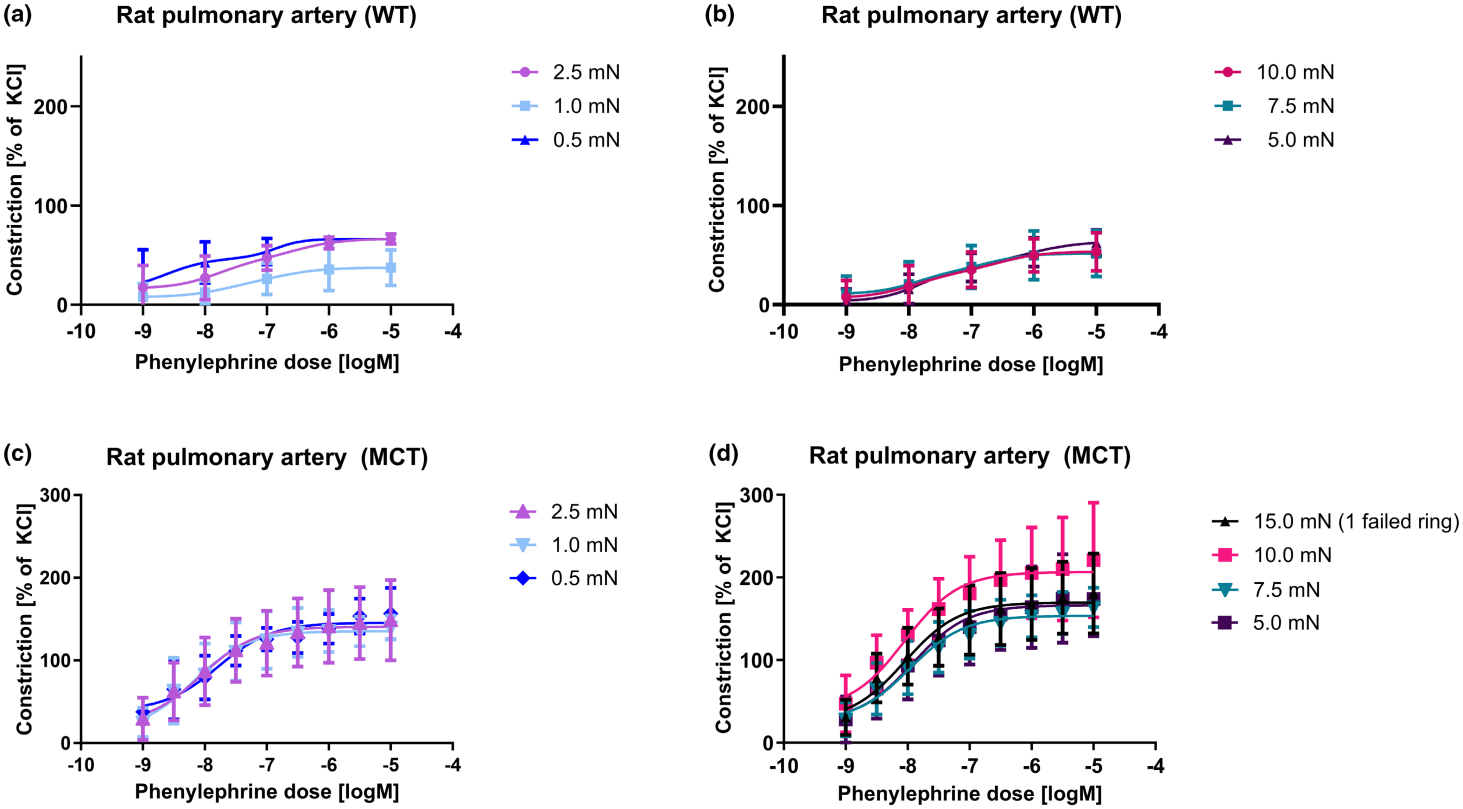
Phenylephrine dose response in rat pulmonary arteries. (a) Dose response to phenylephrine (PE) relative to the contractile response of the vessel to KCl of healthy pulmonary arteries stretched to low levels of initial tension (0.5, 1.0, and 2.5 mN). (b) Dose response to PE of healthy pulmonary arteries stretched to high levels of initial tension (5.0, 7.5, and 10.0 mN). (c) Dose response to PE of pulmonary arteries from animals with PAH stretched to low levels of initial tension (0.5, 1.0, and 2.5 mN), and (d) dose response to of pulmonary arteries from animals with PAH stretched to high levels of initial tension (15.0, 10.0, 7.5, and 5.0 mN).

We next subjected the vessels to increasing concentrations of the α‐adrenoreceptor agonist, phenylephrine (PE), to identify the pre‐stretch condition that yields the maximal dynamic range (Figure 3). Healthy vessels pre‐stretched to 0.5, 1.0, and 2.5 mN (low initial stretch) exhibited a varied response and maximum values (Figure 3a), while, healthy PAs stretched to 5.0, 7.5 and 10.0 mN (high initial stretch) exhibited uniform vasoconstrictor responses (Figure 3b). No statistically significant differences were observed among the maximum normalized constrictions (Supplementary Table S1). Vessels stretched to 0.5 or 1.0 mN had a shallow increase in activity with increasing PE concentrations, thus, the dynamic ranges of vasoreactivity for lower initial tensions were lower than those of the higher tensions (Supplementary Table S1). The dynamic range of vessels from rats with PAH stretched to lower initial tensions (0.5, 1.0, and 2.5 mN) were lower than the dynamic ranges of vessels stretched to higher initial tensions (Supplementary Table S1; Figure 3c). Pulmonary arteries from rats with PAH that were stretched to higher initial tensions (5.0, 7.5, 10.0, and 15.0 mN) exhibited more pronounced responses to PE with higher average maximum normalized constriction values (Supplementary Table S1; Figure 3d). Identical results were obtained when absolute values were analyzed (Supplementary Figure S1).

### 3.5 Initial tension effects on endothelium‐dependent relaxation

**FIGURE 4:**
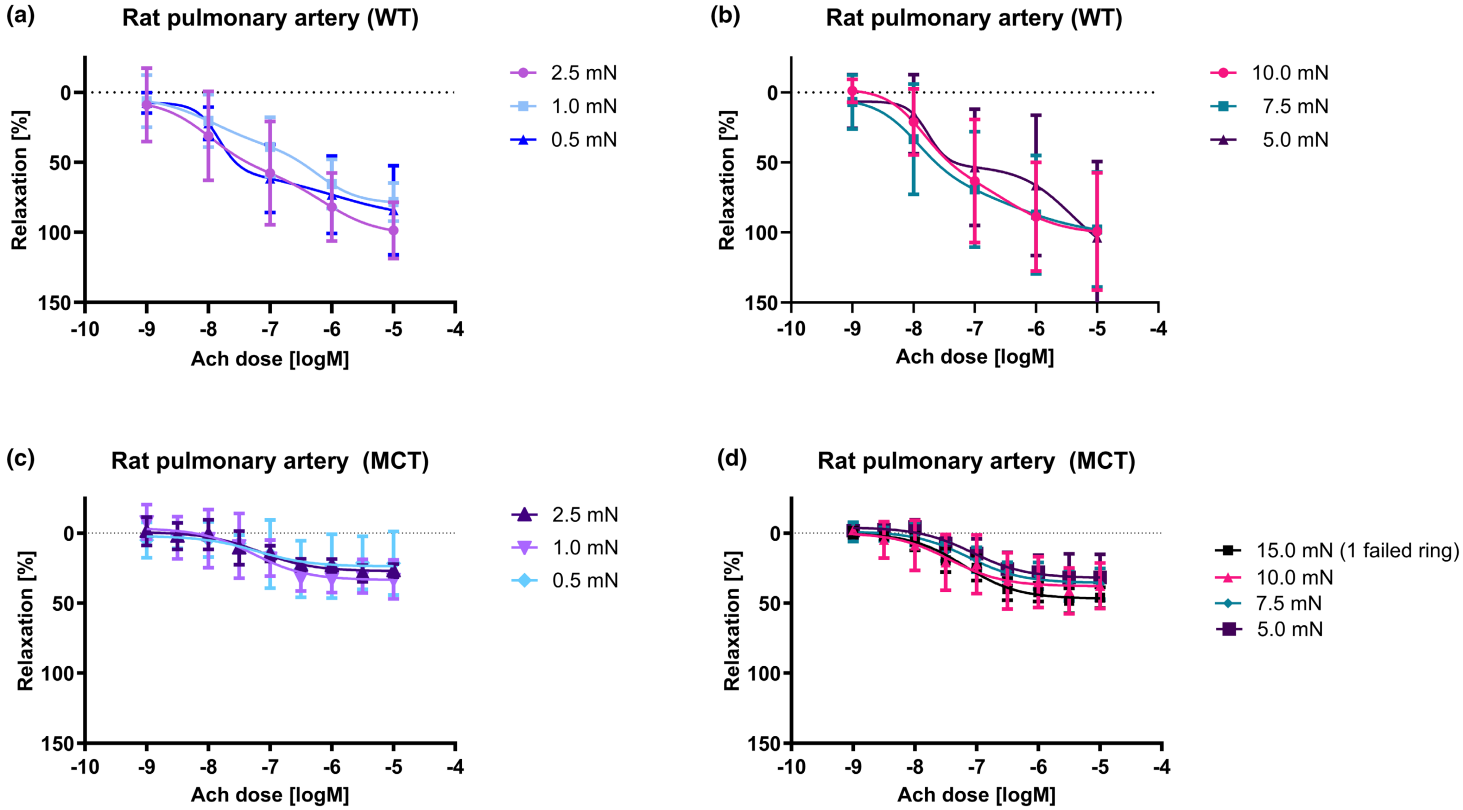
Acetylcholine dose response in rat pulmonary arteries. (a) Dose response to acetylcholine (Ach) of healthy pulmonary arteries stretched to low levels of initial tension (0.5 mN, 1.0 mN, 2.5 mN). (b) Dose response to Ach of healthy pulmonary arteries stretched to high levels of initial tension (5.0 mN, 7.5 mN, 10.0 mN) (c) Dose response to Ach of pulmonary arteries from animals with PAH stretched to low levels of initial tension (0.5 mN, 1.0 mN, 2.5 mN), and (d) dose response to Ach of pulmonary arteries from animals with PAH stretched to high levels of initial tension (15.0 mN, 10.0 mN, 7.5 mN, 5.0 mN).

The endothelium‐mediated relaxation was assessed in vessels pre‐constricted to a similar tone with phenylephrine (10^−6^ M), followed by increasing concentrations of acetylcholine (Ach; 10^−9^–10^−5^ M; Figure 4). For healthy vessels subjected to lower initial tensions (0.5, 1.0, and 2.5 mN), the dynamic range were significantly lower than those of the higher initial tensions (Figures 4a and 4b; Supplementary Table S2). Healthy PAs of higher initial tensions (2.5, 5.0, 7.5, and 10.0 mN) showed higher maximum relaxation compared to lower initial tensions. In addition, while maximal relaxation was similar in the higher initial tension groups (Figure 4b), 7.5 10.0 mN produced more robust, sigmoidal dose–response curves and exhibited the best dynamic range. (Figure 4b; Supplementary Table S2). The endothelium dependent relaxations of vessels from animals with PAH were significantly attenuated compared to healthy PAs (Supplementary Table S2) independent of the initial tension. Vessels from animals with PAH of higher initial tensions (5.0, 7.5, 10.0, and 15.0 mN) exhibited higher average maximum normalized relaxation than MCT vessels of lower initial tensions (0.5, 1.0, and 2.5 mN), with the highest normalized relaxation observed for MCT vessels stretched to 15.0 mN (Figure 4d, Supplementary Table S2). Notably, the 15.0 mN initial tension vessels exhibited the highest dynamic range of 46.87% of the KCl response, indicating a positive correlation between the amount of initial stretch to the agonist stimulated release of nitric oxide (NO), for example.

### 3.6 Initial tension effects on endothelium‐independent relaxation

**FIGURE 5:**
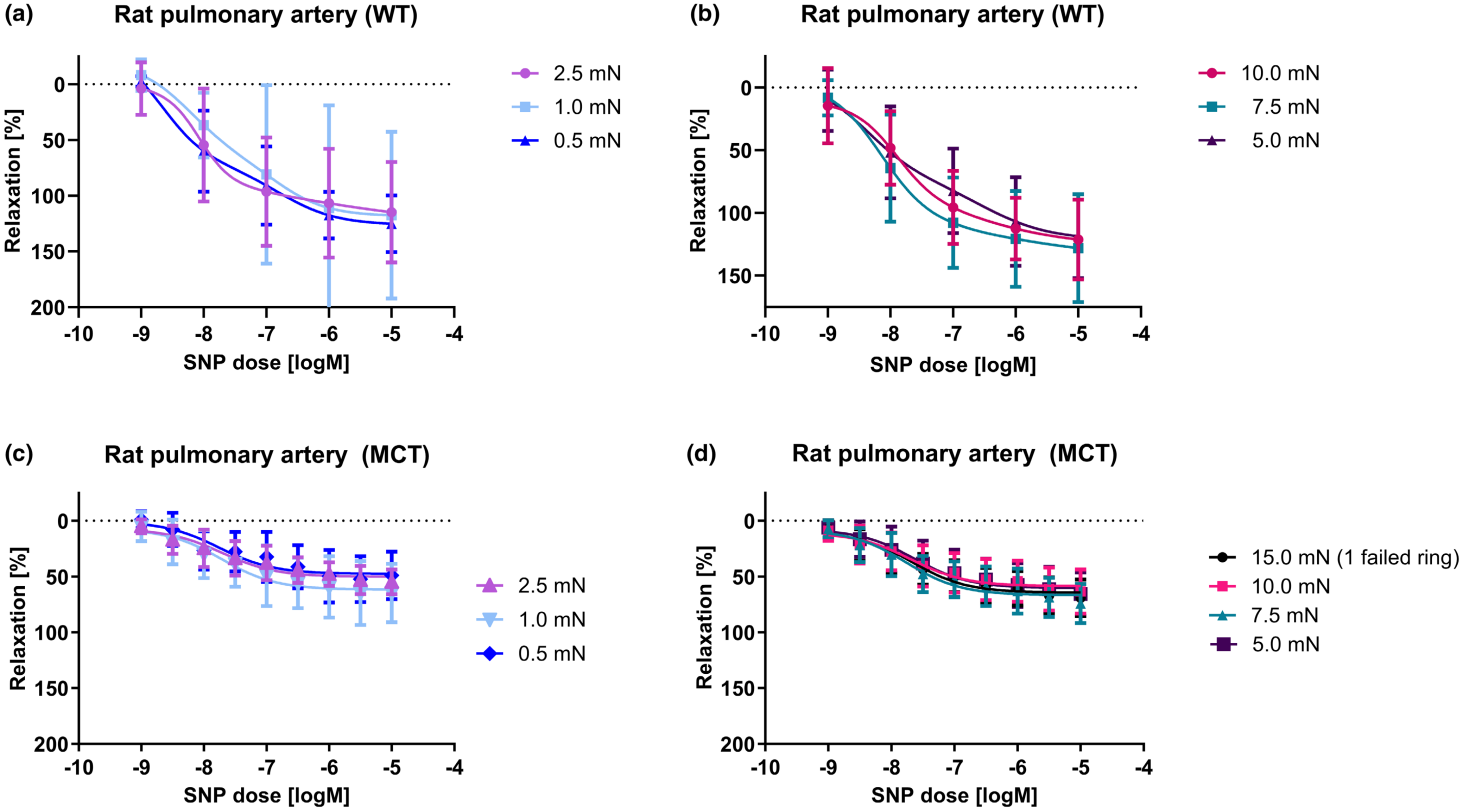
Sodium nitroprusside dose response in rat pulmonary arteries. (a) Dose response to sodium nitroprusside (SNP) of healthy pulmonary arteries stretched to low levels of initial tension (0.5 mN, 1.0 mN, 2.5 mN). (b) Dose response to SNP of healthy pulmonary arteries stretched to high levels of initial tension (5.0 mN, 7.5 mN, 10.0 mN) (C) Dose response to SNP of pulmonary arteries from animals with PAH stretched to low levels of initial tension (0.5 mN, 1.0 mN, 2.5 mN), and (d) dose response to SNP of pulmonary arteries from animals with PAH stretched to high levels of initial tension (15.0 mN, 10.0 mN, 7.5 mN, 5.0 mN).

Finally, we investigated the effect of varying degrees of initial tensions on the endothelium‐independent relaxation of pre‐constricted vessels using sodium nitroprusside (SNP; 10^−9^–10^−5^ M; Figure 5). Maximum relaxation was independent of the pre‐stretch imposed both for vessels of higher initial tensions (5.0, 7.5, and 10.0 mN stretches) and, those of lower initial tensions (0.5, 1.0, and 2.5 mN stretches; Figure 5a,b, Supplementary Table S3). However, the average EC50 values of the higher initial tension groups were lower and more uniform than that of the lower initial tension groups (7.92 ± 0.03 vs. 8.03 ± 0.31). The dynamic ranges of relaxation for both groups were also comparable (Supplementary Table S3). The SNP response was significantly blunted in vessels from animals with PAH compared to healthy controls. However, within the PAH group, we observed a similar trend in relaxation response where PAH vessels of higher initial tension groups (5.0, 7.5, 10.0, and 15.0 mN) consistently exhibited higher average maximum relaxations and higher dynamic ranges than PAH vessels of lower initial tension (0.5, 1.0, and 2.5 mN; Supplementary Table S3).

## 4 DISCUSSION

In this study, we examined the contribution of initial tension to vessel viability and dose‐dependent vasomotor functions to optimize experimental protocols for rat pulmonary artery wire myograph. We used an empirical approach given the low arterial blood pressure in the pulmonary circulation, using IC100 as a guideline. We conclude that a moderate amount of initial tension (7.5 mN) is optimal for pulmonary arteries from healthy animals for wire myograph experiments. These values provided a good dynamic range for vasocontractile and vasodilatory responses, without compromising vessel integrity. Pulmonary arteries from an animal with PAH require higher levels of initial stretch, up to 15.0 mN, given the significant vascular remodeling those vessels have undergone. This is distinct from the values used in prior studies, which range from 1.0 to 6.0 mN because the metric to select the optimal initial tension in this study was based on the dynamic range of vasoconstriction and vasorelaxation responses to various agonists and antagonists and not solely to the initial response to KCl.

The type, size, and location of arteries are central elements that influence contractile vascular activity and resistance (Ko et al., 2010). Arterioles (100–500 μm in diameter) contribute greatly to flow and blood pressure regulation in vivo (Ko et al., 2010). While large vessels contribute less to total vascular resistance, the direct coupling between the ventricles and the large arteries makes it important to study the vasoreactivity of those large, compliance vessels, particularly in hypertension, aging, and atherosclerosis, where functional responses of the large vessels are compromised due to increased mechanical stiffness (Sun & Chan, 2018). Focusing on the pulmonary circulation, wire myograph experiments using segments of vessels from different locations within the main pulmonary artery have provided significant qualitative insight into different disease models (Ko et al., 2010). Consistent with prior research, we elected to isolate the first branches of the main pulmonary artery (the left and right pulmonary artery, Figure 1a), which were more conducive to wire myography experiments given their size relative to the pins (Mulvany & Aalkjaer, 1990). Both were tested to determine differences between the two branches. However, we noted no differences in the pre‐stretch required for the left and right branches despite the significant difference in size of these vessels (Figure 1 and Supplementary Figure S2). Thus, the results of wire myography are independent of which branch is selected in a particular study, which is consistent with prior studies (Ramachandra & Humphrey, 2019).

Using different degrees of pre‐stretch demonstrated that the optimal pre‐stretch for healthy pulmonary arteries of rats is at relatively high levels (7.5 mN for vessel segments of internal radius 2 mm and length 2 mm). This was unexpected given that the pulmonary arteries are a low‐pressure system and that the established pre‐stretch in rat aortas, that experience significantly higher mean pressure in vivo, is 15.0 mN (15.0 mN for vessel segments of internal radius 2 mm and length 2 mm). Pulmonary arteries isolated from animals with PAH, however, required a 15.0 mN pre‐stretch, roughly twice that of the healthy vessels. This is consistent with the ~two‐fold increase in pulmonary pressures in PAH. The incremental values of pre‐stretch to achieve the final tension were determined in such manner as to minimize the number of pre‐stretch steps required to achieve the intended final tension and prevent the vessels from being under extreme mechanical stress for a long duration of time. The ranges of initial pre‐stretch levels between 7.5 and 10.0 mN, was determined as an area where additional pre‐stretch levels could be tested to identify the optimal value for each individual application. Further investigation into determining those increments of pre‐stretch could effectively minimize the mechanical stress imposed on the vessel segments during the pre‐stretch and might change the ability of the vessel to withstand higher levels of pre‐stretch.

Interestingly, while initial tension contributed to the difference observed in dose–response results acquired in all three of the pharmaceutical agents tested (phenylephrine, acetylcholine, and sodium nitroprusside), the highest impact of initial tension was noted on the endothelium‐dependent relaxation induced by Ach (Figure 4). Ach produced stable and consistent sigmoidal dose–response curves for vessel segments stretched to 7.5 and 10.0 mN, while SNP, which relaxes vessels independent of NO, produced stable and consistent sigmoidal curves for vessel segments of initial tensions ranging for a broader range of pre‐stretch (1.0 to 10.0 mN). Lower tension values likely did not cause relaxation as (1) the dynamic range to measure Ach induced relaxation was insufficient, and (2) the endothelium required a minimal level of stretch to function effectively. The latter can be explained by the vascular endothelium being exposed to both cyclic stretch due to the pulsatile beating of the heart and to wall shear stress due to blood flow—thus, the endothelium is highly sensitive to mechanical force (Thorin & Thorin‐Trescases, 2009). As acetylcholine response requires the vascular endothelium to be structurally and physiologically intact, it can be reasoned that moderate levels of initial tensions more closely and accurately mimic the in‐vivo parameters of pulmonary arteries with regard to mechanical signaling than high (15.0 mN) or low (1.0, 2.5 and 5.0 mN) values of initial tension–and this response is shifted upwards in vessels that have undergone remodeling in PAH. Therefore, in order to account for the limitations of endothelium‐dependent relaxation by Ach, it is crucial that the range of initial tensions must also be limited to the range where vessels are most reactive and show robust endothelium‐dependent relaxations in isometric tension experiments involving a wide variety of diverse vasodilators and vasoconstrictors.

Expanding the data in healthy animals, dose‐responses of rat we interrogated the validity of the optimized protocol in a rat PAH model, and observed significantly different responses to all vasoactive agents. This effect can be attributed to the severe vascular remodeling in PAH caused by growth and proliferation of smooth muscle cells, ultimately resulting in changes in vasomotor functions. While healthy pulmonary arteries produced consistent, sigmoidal dose‐responses when stretched to a moderate initial tension of 7.5 mN, we observed similar results in 15.0 mN initial tension groups for MCT administered rats, reflecting the higher pulmonary artery wall tension in the PAH model in‐vivo. These observations allowed us to characterize the endothelial and smooth muscle contribution to increased vessel stiffness and wall tension as a result of PAH. Wall tension analysis in relation to the applied initial tension, not only provides insight into the ex‐vivo characterization of PAH vessel function but also a standard to which future wire myograph experiments can be planned depending on the vascular stiffness of the diseased vessels in different pathological states.

Advantages of this technique are the ability to accurately model and mimic physiological systems of different pathological states, and to further observe and measure changes in vessel contractility. This is a valuable tool to investigate the development of, and the clinical implications of pulmonary vascular diseases, such as PAH. Furthermore, multiple wire myograph units can be utilized to perform a comparative analysis of the effect that different pharmaceutical agents have on rat PAs of identical pathological states in order to compare the efficacy of targeting different signaling pathways in the development of PAH. We suggest that the optimal values of pre‐stretch obtained in this study as a starting point to be expanded and reinforced by application to various strain of rats, and with different models of pulmonary diseases.

## AUTHOR CONTRIBUTIONS

RC, RN, SJ, and SK carried out the experiment. JS, BW, and HW carried out the MCT rat experiments. RC and JS developed the theory and performed the computations. LS and JS verified the analytical methods. RC wrote the manuscript with support from LS and JS. All authors discussed the results and contributed to the final manuscript. LS and JS conceived the original idea, and supervised the project.

## FUNDING INFORMATION

This work was supported by a NHLBI grant R01HL148112 (L.S.), two Stimulating and Advancing ACCM Research (StAAR) grants from the Department of Anesthesiology and Critical Care Medicine, Johns Hopkins University (L.S and J.S.), and a NHLBI grant K08HL145132 (J.S.).

## CONFLICT OF INTEREST STATEMENT

The authors have no financial conflict of interest to disclose.

## ETHICS STATEMENT

The present study followed international, national, and institutional guidelines for humane animal treatment and complied with relevant legislation. All animal protocols were approved by the Johns Hopkins Animal Care and Use Committee.

## Supporting information


Data S1.

## Data Availability

The data that support the findings of this study are available from the corresponding author upon reasonable request.
