# Supplementary material for: Optimization of resting tension for wire myography in male rat pulmonary arteries
Source: Physiol Rep. 2024 Jan 11;12(1):e15911. doi: 10.14814/phy2.15911 (PMC10784191; doi:10.14814/phy2.15911)
Supplement: Supplementary file 1 — Data S1. [file PHY2-12-e15911-s001.docx]

**SUPPORTING INFORMATION**

**Optimization of wire myography protocol for rat pulmonary arteries**

Rira Choi^1,3,a^, Roshini Narayanan^2^, Sandeep Jandu^3^, William Savage^1^, Sara Kang^3^, Bulouere Wodu^3^, Kavitha Nandakumar^3^, Lakshmi Santhanam^1,2,3^, Jochen Steppan^3*^

^1^ Department of Chemical and Biomolecular Engineering, Johns Hopkins University, Baltimore, Maryland, United States of America

^2^ Department of Biomedical Engineering, Johns Hopkins University, Baltimore, Maryland, United States of America

^3^ Department of Anesthesiology and Critical Care Medicine, Johns Hopkins School of Medicine, Maryland, United States of America

^a^ Current Address: Department of Biomedical Engineering, Yale University, New Haven, Connecticut, United States of America

**Supplementary Table 1: Phenylephrine Dose Response. Supplementary** Table 1 describes the phenylephrine dose response results of both WT and MCT animals with their maximum normalized constriction values and their pEC50. WT: wildtype, MCT: monocrotaline

|  |  | 0.5 mN | 1.0 mN | 2.5 mN | 5.0 mN | 7.5 mN | 10.0 mN | 15.0 mN |
| --- | --- | --- | --- | --- | --- | --- | --- | --- |
| WT  (n=22) | **Maximum Normalized Constriction** | 66.34  ±2.48 | 37.28  ±8.99 | 66.32  ±2.90 | 62.35  ±4.63 | 51.52  ±7.81 | 53.37  ±6.47 | - |
|  | **pEC50** | 8.64 | 7.64 | 7.82 | 7.83 | 7.88 | 7.92 | - |
|  | **Dynamic Range** | 65.39 ±11.97 | 27.55 ±5.07 | 47.64 ±5.74 | 51.52 ±2.75 | 40.70 ±2.18 | 48.29 ±2.10 | - |
| MCT  (n=18) | **Maximum Normalized Constriction** | 145.7  ±5.21 | 135.5  ±5.87 | 140.8  ±8.18 | 166.3  ±8.46 | 153.6  ±5.45 | 206.6  ±8.53 | 169.7  ±8.1 |
|  | **pEC 50** | 7.79 | 8.18 | 8.06 | 7.92 | 7.97 | 8.05 | 8.05 |
|  | **Dynamic Range** | 107.0 ±5.25 | 121.3 ±8.97 | 118.1 ±10.76 | 140.4 ±8.75 | 128.0 ±5.93 | 167.1 ±8.53 | 143.6 ±10.50 |

**Supplementary Table 2: Acetylcholine Dose Response.** Supplementary Table 2 depicts the acetylcholine dose response results of both WT and MCT animals with their maximum normalized constriction values and their pEC50. WT: wildtype, MCT: monocrotaline

|  |  | 0.5 mN | | 1.0 mN | 2.5 mN | 5.0 mN | 7.5 mN | 10.0 mN | 15.0 mN |
| --- | --- | --- | --- | --- | --- | --- | --- | --- | --- |
| WT  (n=22) | **Maximum Normalized Relaxation** | | 84.13  ±18.27 | 78.24  ±4.48 | 98.48  ±6.66 | 103.65  ±16.37 | 97.82  ±12.36 | 99.25  ±13.21 | - |
|  | **pEC50** | | 7.83 | 7.93 | 7.99 | 7.76 | 7.94 | 7.85 | - |
|  | **Dynamic Range** | | 75.01 ±8.84 | 66.24 ±2.37 | 79.62 ±3.76 | 84.81 ±3.84 | 82.94 ±4.36 | 95.90 ±3.57 | - |
| MCT  (n=18) | **Maximum Normalized Relaxation** | | 23.65 ±5.44 | 33.4 ±4.15 | 27.03 ±1.91 | 31.87 ±2.68 | 35.45 ±1.9 | 37.81 ±3.11 | 46.73 ±1.60 |
|  | **pEC50** | | 7.21 | 7.27 | 7.18 | 7.07 | 7.13 | 7.44 | 7.20 |
|  | **Dynamic Range** | | 21.75 ±4.88 | 37.12 ±3.31 | 27.94±1.19 | 36.20 ±1.59 | 36.86 ±1.09 | 38.13 ±1.82 | 46.87 ±0.93 |

**Supplementary Table 3: Sodium Nitroprusside Dose Response.** Supplementary Table 3 describes the sodium nitroprusside dose response results of both WT and MCT animals with their maximum normalized constriction values and their pEC50. WT: wildtype, MCT: monocrotaline

|  |  | 0.5 mN | 1.0 mN | 2.5 mN | 5.0 mN | 7.5 mN | 10.0 mN | 15.0 mN |
| --- | --- | --- | --- | --- | --- | --- | --- | --- |
| WT  (n=22) | **Maximum Normalized Relaxation** | 125.03  ±17.74 | 117.40  ±30.49 | 114.73  ±16.92 | 118.48  ±12.61 | 128.00  ±17.51 | 121.15  ±11.21 | - |
|  | **pEC50** | 8.67* | 8.24 | 8.07 | 8.38 | 8.15 | 7.91 | - |
|  | **Dynamic Range** | 131.20 ±19.74 | 123.09 ±14.48 | 117.01 ±9.63 | 102.04 ±6.54 | 127.24 ±9.23 | 106.28 ±4.86 |  |
| MCT  (n=18) | **Maximum Normalized Relaxation** | 47.35 ±4.24 | 61.51 ±4.76 | 50.00 ±2.68 | 60.15 ±3.57 | 66.48 ±3.20 | 58.4 ±3.00 | 64.22 ±2.92 |
|  | **pEC50** | 7.67 | 7.69 | 7.77 | 7.64 | 7.71 | 7.72 | 7.76 |
|  | **Dynamic Range** | 46.97 ±4.91 | 54.55 ±4.12 | 42.96 ±2.30 | 52.75 ±2.71 | 58.56 ±2.67 | 47.89 ±2.08 | 54.59 ±2.26 |

**Supplementary Figure 1: Absolute phenylephrine dose response in rat pulmonary arteries**

A) Dose response to phenylephrine (PE) of healthy pulmonary arteries stretched to low levels of initial tension (0.5, 1.0, and 2.5 mN), B) Dose response to PE of healthy pulmonary arteries stretched to high levels of initial tension (5.0, 7.5, and 10.0 mN), C) Dose response to PE of pulmonary arteries from animals with PAH stretched to low levels of initial tension (0.5, 1.0, and 2.5 mN), and D) Dose response to of pulmonary arteries from animals with PAH stretched to high levels of initial tension (15.0, 10.0, 7.5, and 5.0 mN).


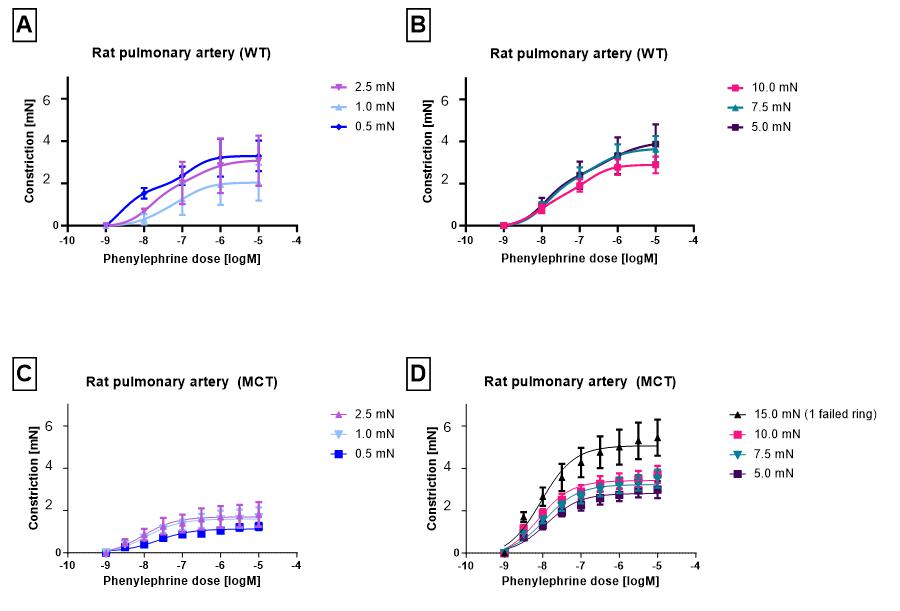


**Supplementary Figure 2: Maximum contraction response of the left and right pulmonary artery**

A) This figure demonstrates the maximum contractile response of the left pulmonary artery stretched to a different initial tension. B) This figure demonstrates the maximum contractile response of the right pulmonary artery stretched to a different initial tension.
